# Dynamics‐based characterization and classification of biodiversity indicators

**DOI:** 10.1002/ece3.10271

**Published:** 2023-07-06

**Authors:** Yuri Otomo, Reiji Masuda, Yutaka Osada, Kazutaka Kawatsu, Michio Kondoh

**Affiliations:** ^1^ Graduate School of Life Sciences Tohoku University Sendai Japan; ^2^ Maizuru Fisheries Research Station Kyoto University Kyoto Japan

**Keywords:** biodiversity indicators, coastal fish community, dynamics, nonlinear time‐series analysis

## Abstract

Various biodiversity indicators, such as species richness, total abundance, and species diversity indices, have been developed to capture the state of ecological communities over space and time. As biodiversity is a multifaceted concept, it is important to understand the dimension of biodiversity reflected by each indicator for successful conservation and management. Here we utilized the responsiveness of biodiversity indicators' dynamics to environmental changes (i.e., environmental responsiveness) as a signature of the dimension of biodiversity. We present a method for characterizing and classifying biodiversity indicators according to environmental responsiveness and apply the methodology to monitoring data for a marine fish community under intermittent anthropogenic warm water discharge. Our analysis showed that 10 biodiversity indicators can be classified into three super‐groups based on the dimension of biodiversity that is reflected. Group I (species richness and community mean of latitudinal center of distribution (cCOD)) showed the greatest robustness to temperature changes; Group II (species diversity and total abundance) showed an abrupt change in the middle of the monitoring period, presumably due to a change in temperature; Group III (species evenness) exhibited the highest sensitivity to environmental changes, including temperature. These results had several ecological implications. First, the responsiveness of species diversity and species evenness to temperature changes might be related to changes in the species abundance distribution. Second, the similar environmental responsiveness of species richness and cCOD implies that fish migration from lower latitudes is a major driver of species compositional changes. The study methodology may be useful in selecting appropriate indicators for efficient biodiversity monitoring.

## Graphical abstract

Understanding the dimensions biodiversity indicators for indicator selection is an important issue in ecology. This study focused on the environmental responsiveness of biodiversity indicators' dynamics as a signature of the dimension of biodiversity. We evaluated the environmental responsiveness of 10 biodiversity indicators time series derived from actual fish community data and found that the 10 biodiversity indicators were classified into three super‐groups.

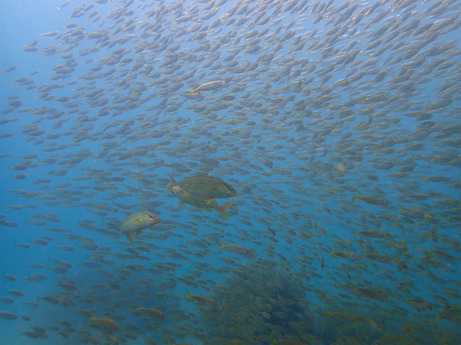

## 1 INTRODUCTION

Biological diversity (i.e., biodiversity) is a multifaceted concept (Giller et al., 2004; Purvis & Hector, 2000) and many biodiversity indicators (e.g., species richness and species evenness) have been proposed. Different indicators reflect different aspects of biodiversity (biodiversity dimensions) and may therefore show different responses to environmental changes. For example, a study of a benthic community have shown that species richness and total biomass, but not species evenness, reflect the impact of bottom trawling (Hiddink et al., 2020). Therefore, it is essential for researchers, managers, and policy makers to select appropriate biodiversity indicators that capture the dimensions of interest. Given the recent increase in anthropogenic pressures and associated changes in biodiversity, it is important to understand the dimension of biodiversity reflected by each biodiversity indicator for successful conservation and management.

A number of studies have explored the similarity among biodiversity indicators based on correlations among time‐independent (or “snapshot”) community data (Heino et al., 2008; Lyashevska & Farnsworth, 2012; Morris et al., 2014; Soininen et al., 2012; Stevens & Tello, 2018; Wilsey et al., 2005). For example, Lyashevska and Farnsworth (2012) examined correlations among 19 biodiversity indicators and found that structural complexity (e.g., species diversity), functional diversity, and species richness each reflect a different dimension of biodiversity. However, the responses of biodiversity indicators to environmental change can be highly condition dependent and therefore a lack of a correlation may not indicate dissimilarity in related dimensions. In fact, there is non‐stationarity in similarity and correlations (De Benedictis, 1973; Morris et al., 2014; Soininen et al., 2012; Stirling & Wilsey, 2001) among biodiversity indicators, further suggesting that the response of biodiversity indicators to environmental changes is condition dependent.

Here, we propose a dynamics‐based methodology to identify the similarity in biodiversity dimensions reflected by various indicators and apply it to real‐world fish community monitoring data. In particular, we use environmental responsiveness (i.e., responsiveness of the dynamics of biodiversity indicators to environmental change) to determine the biodiversity dimension reflected by various indicators. Consider a biodiversity indicator X related to a biodiversity dimension Y. Environmental changes can alter not only the state but also the dynamics (rules of state transitions) of ecosystems (Becks et al., 2005). Therefore, if an environmental change induces a change in the dynamics of dimension Y, it should also induce changes in the dynamics of indicator X. Thus, in a temporally varying environment, the dynamics of indicators reflecting a similar dimension would show similar temporal patterns. The similarity in environmental responsiveness between indicators, measured based on the timing of changes in dynamics, can be used to evaluate similarity in biodiversity dimensions captured by the indicators. This methodology can be applied even when the response (e.g., sign) of the indicator value to an environmental change is condition dependent and there is no correlation between indicators related to a similar biodiversity dimension.

In this study, we examined the similarity in environmental responsiveness among 10 biodiversity indicators based on 7‐year monitoring data for coastal fish communities exposed to anthropogenic changes in water temperature due to the intermittent activation and inactivation of a nuclear power plant (Masuda, 2020). To assess the similarity in environmental responsiveness, nonlinear prediction‐based methodology (Schreiber, 1997) was applied to compiled time‐series data for each biodiversity indicator. We found three groups of biodiversity indicators with similar environmental responsiveness in dynamics. Finally, we discuss how the methodology can be used to select appropriate biodiversity indicators for conservation and management purposes.

## 2 MATERIALS AND METHODS

### 2.1 Time‐series data for a fish community

A long‐term monitoring dataset for a marine fish community on the coast of Uchiura Bay (35°32′N, 135°30′E) was used. The monitoring area was located 2 km from the discharge outlet of the Takahama nuclear power plant (NPP). The Takahama NPP started operation in 1974 and was shut down for two periods: 4 years from February 2012 to January 2016, and 14 months from March 2016 to May 2017 (The Kansai Electric Power, 2021). During operation, the Takahama NPP drains a maximum thermal discharge of 238 m^3^/s, which is 7°C higher than the water temperature in the natural environment (Kokaji, 1995). As a result, the temperature increase in the survey area due to the NPP operation is approximately 2°C (Masuda, 2020).

Abundance data for each fish species were obtained by direct visual underwater surveys, covering an area of approximately 1200 m^2^ (2 m wide by 600 m long). These surveys were conducted once a month from January 18, 2012 to April 26, 2019. The fish identification procedure followed that described by Nakabo (2013). The survey method was previously described by Masuda (2020).

**FIGURE 1:**
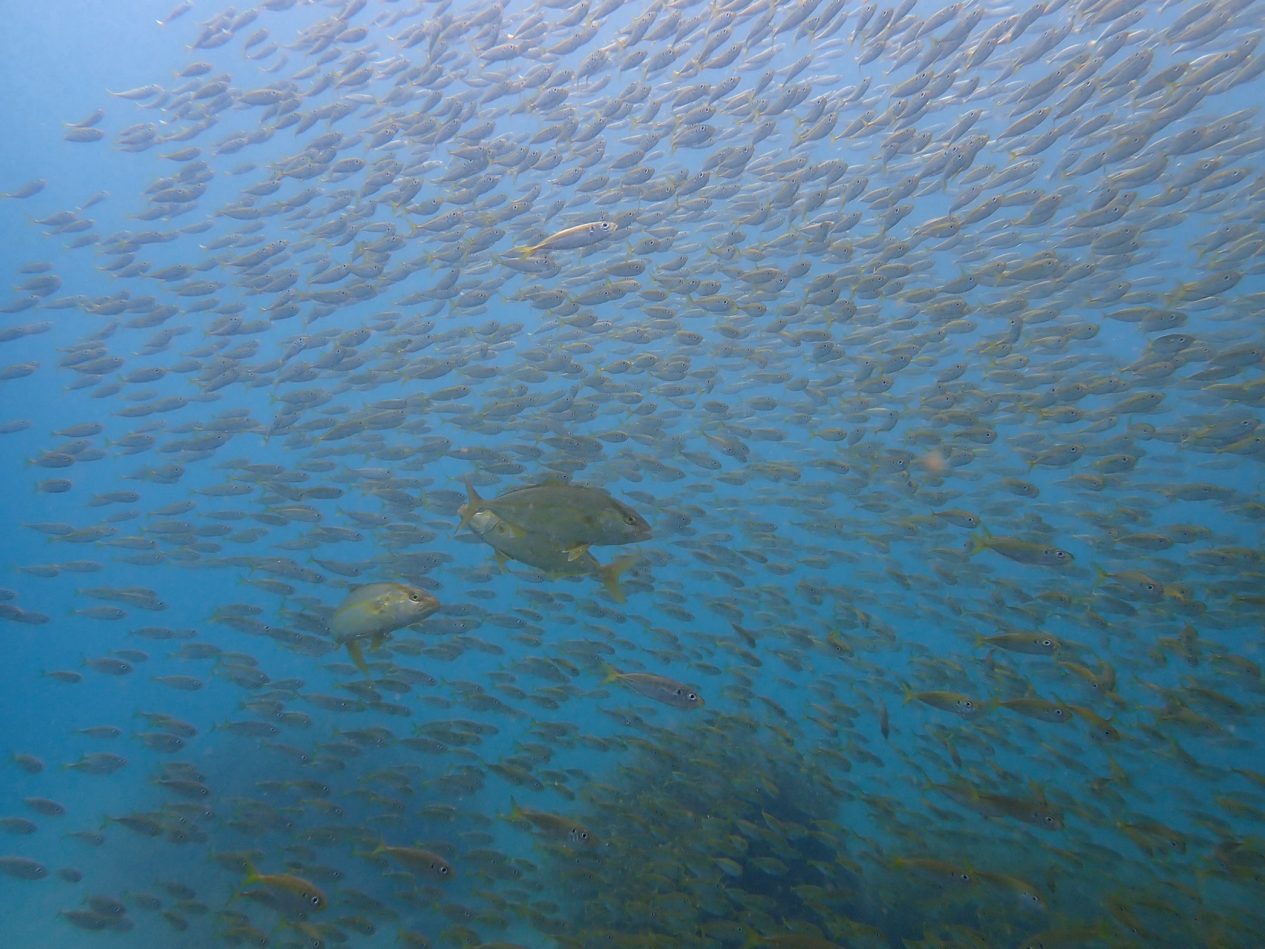
Photograph of fish (*Trachurus japonicus* and *Seriola dumerili*) in the survey area. This photograph was taken by Reiji Masuda at the survey site on September 13, 2021.

**FIGURE 2:**
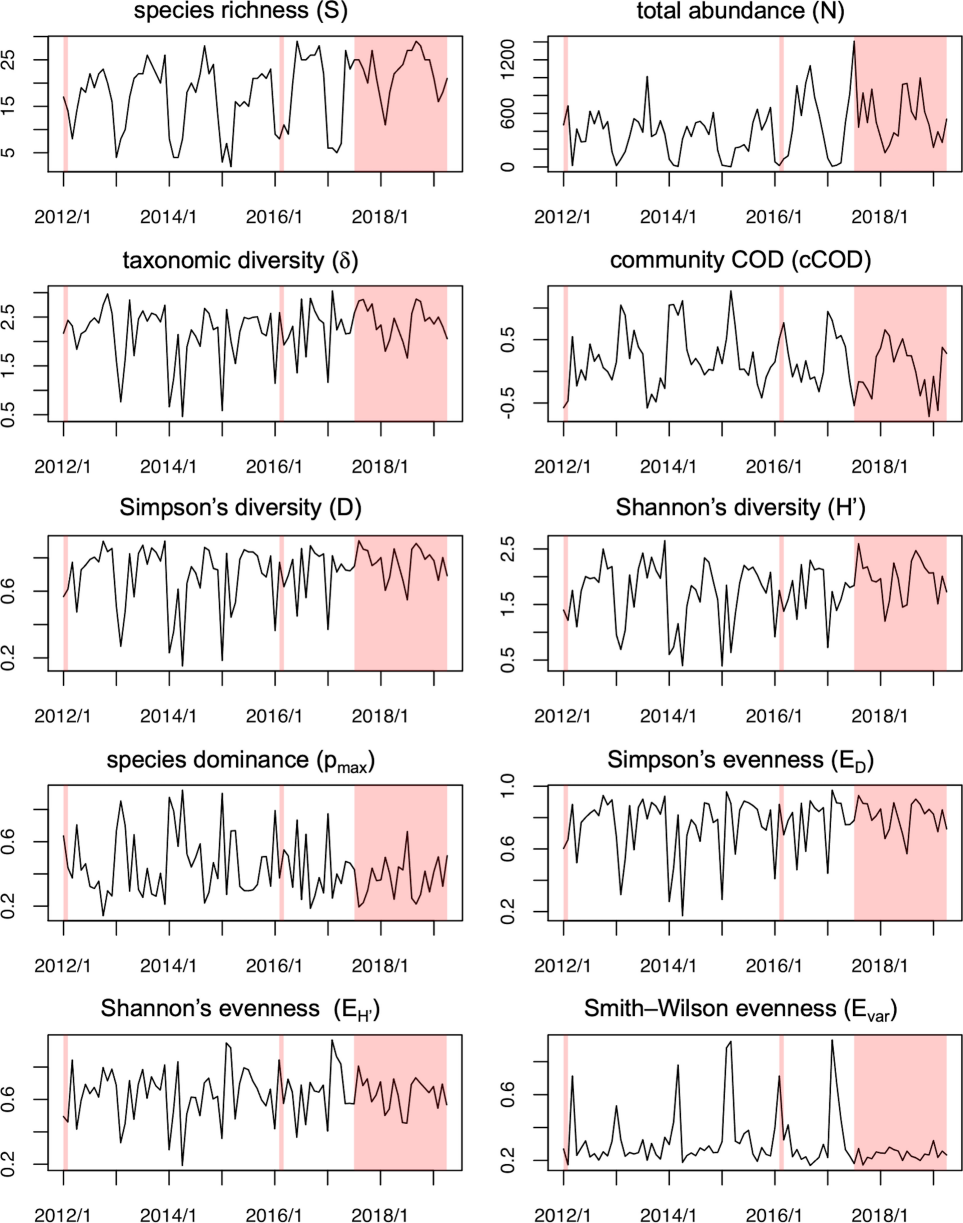
Time‐series data for 10 biodiversity indicators. The horizontal axis shows the time point of each survey and the vertical axis shows the value of each indicator. The red shaded region indicates the period when the NPP was active.

Data were obtained at 88 time points over 7 years. A total of 95 fish species were recorded during the survey periods (Figure 1: Examples of fish species observed in the survey). Using time‐series data, 10 biodiversity indicators were calculated for each survey, including species richness (i.e., number of species), relative abundance of species, and differences in fish taxonomy and geographic distribution (Table 1; actual temporal variation in 10 biodiversity indicators is shown in Figure 2).

**TABLE 1:** Equations used to calculate biodiversity indicators.

Metrics	Equation or explanation	Reference
Species richness (*S*)	Number of fish species observed during each survey	–
Total abundance (*N*)	Total number of fish of all species observed in each survey	–
Species dominance (*p*_max_)	$p_{\max}=\max p_{i}$	Berger and Parker (1970)
Simpson's species diversity (*D*)	$1-\sum_{i}^{S}p_{i}^{2}$	Simpson (1949)
Shannon's species diversity (*H′*)	$-\sum_{i}^{S}p_{i}\;\ln\;p_{i}$	Shannon (1948)
Simpson's species evenness (*E*_D_)	$\frac{\mathrm{DS}}{S-1}$	Simpson (1949)
Shannon's species evenness (*E*_H″_)	$\frac{H^{\prime }}{\ln S}$	Shannon (1948)
Smith–Wilson's species evenness (*E*_var_)	$1-\frac{2}{\pi }\arctan\left(\frac{1}{S}\sum_{i}^{S}{\left(\ln\left(n_{i}\right)-\frac{1}{S}\sum_{j}^{S}\ln\left(n_{j}\right)\right)}^{2}\right)$	Smith and Wilson (1996)
Taxonomic diversity (*δ*)	$\frac{\sum_{i=1}^{S}\sum_{i<j}^{S}w_{\mathrm{ij}}n_{i}n_{j}}{\sum_{i=1}^{S}\sum_{i<j}^{S}n_{i}n_{j}+\sum_{i=1}^{S}n_{i}\left(n_{i}-1\right)/2}$	Warwick and Clarke (1995)
Community mean of COD (cCOD)	$\text{cCOD}=\sum_{i}^{S}p_{i}\;{\mathrm{COD}}_{i}$	–

*Note*: $p_{i}$ is the proportion of the *i*‐th fish species in the total number of individuals observed; $n_{i}$ is the number of individuals of the *i*‐th species observed; *S* is number of species; $w_{\mathrm{ij}}$ is the taxonomic relatedness between the *i*‐ and *j*‐th species (set to 1, 2, 3, and 4 for species pairs within the same genus, within the same family, within the same order, and others, respectively); and ${\mathrm{COD}}_{i}$ is the latitudinal center of distribution (COD) in the Northern Hemisphere of the *i*‐th species (Masuda, 2020; Nakabo, 2013), standardized using the mean and variance of the COD of all species observed during the survey.

### 2.2 Evaluating dynamics of biodiversity indicators

The dynamics change of each biodiversity indicator was assessed using a time‐series analysis called nonlinear mutual prediction (Schreiber, 1997). We assume that biodiversity indicator X and its dynamics may change over time. We can detect a change in dynamics by using a prediction model. Suppose we have a model that accurately captures the dynamics and predicts the temporal change of X over a short time period *T*_A_. If the dynamical rule in another time period *T*_B_ is the same as that in *T*_A_, then the model should perform well in period *T*_B_. Otherwise, if the dynamics changed in *T*_B_, we would not be able to predict the temporal change of X in period *T*_B_ with the prediction model trained with the dynamics in *T*_A_. We used a nonlinear‐prediction method, simplex projection (Sugihara & May, 1990). This approach is based on Takens' theorem (Takens, 1981), which proves that the dynamical properties of a high‐dimensional nonlinear system can be recovered in the lagged coordinate space of a single time series (i.e., state space reconstruction) and allows us to analyze nonlinear dynamics arising from complex interactions of nonequilibrium communities (Chang et al., 2017; Sugihara & May, 1990).

**FIGURE 3:**
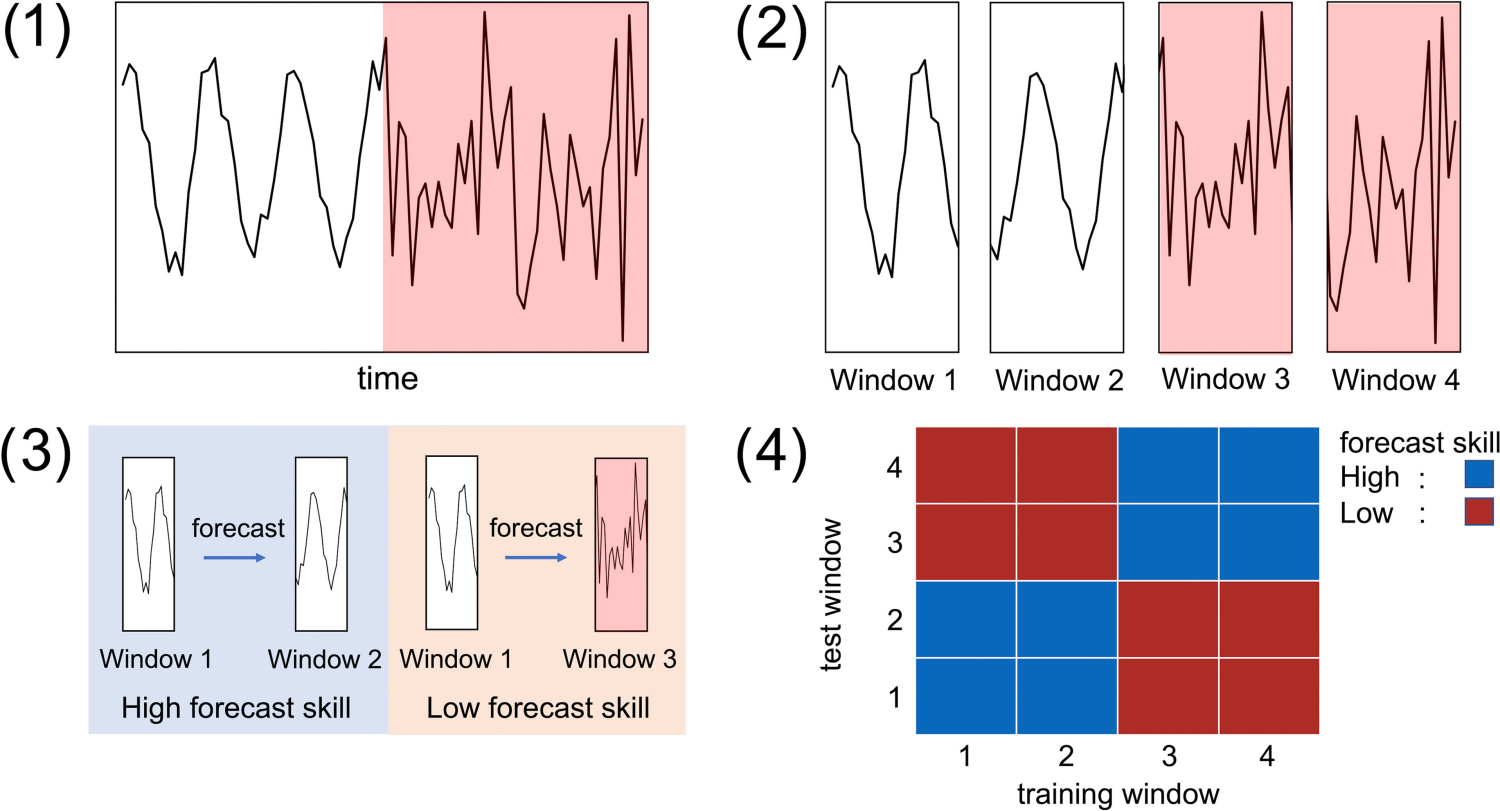
Basic concept of using nonlinear prediction to detect changes in dynamics. In the time series shown in (1), the dynamics differ between the first and second halves. The time series is divided into four windows as shown in (2), and nonlinear prediction is conducted between the windows as shown in (3). The obtained profile (4) indicates that the dynamics changed at the time point around the boundary between Window 2 and 3.

The procedure for nonlinear mutual prediction in our study was as follows. First, 41 sliding windows (i.e., a subset of time‐series data) consisting of 48 consecutive time points were created from the full time‐series of a focal biodiversity indicator. The size of sliding windows was selected by considering the trade‐off between prediction skill and sensitivity for detecting the timing of changes in dynamics (see Appendix S1), particularly the self‐prediction skill of each window and sensitivity for detecting the time points at which mutual prediction becomes impossible (see Figure 3). Second, mutual predictions for all sliding windows derived from the dynamics of each indicator were performed by simplex projection. Specifically, a prediction model was trained based on one sliding window and its prediction skill was tested using other sliding windows. Root mean squared error (RMSE) between the predictions and actual observed values was used as a measure of the prediction skill. Because the prediction skill of simplex projection depends on the choice of embedding dimension (i.e., the number of delay coordinates needed to reconstruct the state space), we searched for the optimal embedding dimension by maximizing the self‐prediction skill of the predictor window. To compare the prediction skill between time series with different variances, we standardized RMSEs as follows:

$$s{\text{RMSE}}_{\mathrm{ij}}=\frac{{\text{RMSE}}_{\mathrm{ij}}}{{\mathrm{sd}}_{j}},$$

where ${\text{RMSE}}_{\mathrm{ij}}$ is the prediction skill using the *i*‐th sliding window as training data and the *j*‐th sliding window as test data, and ${\mathrm{sd}}_{j}$ is the standard deviation of the *j*‐th sliding window. Since ${\mathrm{sd}}_{j}$ can be interpreted as the expected RMSE of an untrained model (i.e., a prediction model that uses the mean of the test data as the prediction value), we assumed that ${\text{sRMSE}}_{\mathrm{ij}}\geq 1$ is a criterion for deterministic prediction failure. Finally, we obtained a 41 × 41 matrix from the results of mutual predictions (i.e., mutual prediction matrix).

### 2.3 Checking the determinism and nonlinearity of biodiversity indicators

Before investigating the similarity in environmental responsiveness among biodiversity indicators, we checked two important dynamical properties, determinism and nonlinearity, of each biodiversity indicator by a self‐prediction analysis. Determinism was quantified by whether $s{\text{RMSE}}_{\mathrm{ii}}<1$ or not. In general, there are two possible situations of ${\text{sRMSE}}_{\mathrm{ii}}\geq 1$, the system is completely stochastic or dynamical mechanisms change within the target sliding window. Thus, we can consider the proportion of 41 sliding windows with $s{\text{RMSE}}_{\mathrm{ii}}<1$ as a measure of determinism. Nonlinearity was quantified by the parameter *θ* of the regularized S‐map (Cenci et al., 2019). Values of *θ* > 0 are associated with the sensitive dependence of the nonlinear system on the initial conditions (Sugihara, 1994). We calculated the parameter *θ* for all 41 sliding windows and performed Dunnett's significance test with the null hypothesis *θ* = 0.0.

### 2.4 Similarity in environmental responsiveness among biodiversity indicators

We measured the similarity in environmental responsiveness among biodiversity indicators using mutual prediction matrices. For simplicity, we calculated the Euclidean distances between mutual prediction matrices of each pair of biodiversity indicators. Based on these Euclidean distances, we classified biodiversity indicators using hierarchical clustering with Ward's method. The significance of the clustering was evaluated by a permutation test with the similarity profile routine (SIMPROF; Clarke et al., 2008), with a significance level of 0.01.

All analyzes were performed using R version 4.0.2 (R Core Team, 2020) and the R packages adiv (version 2.0.1, for the calculation of species diversity and evenness indices), rEDM (version 0.7.5, for the simplex projection analysis), glmnet (version 4.1‐3, for the regularized S‐map analysis), SimComp (version 3.3, for Dunnett's tests), and clustsig (version 1.1, for the permutation test with SIMPROF).

## 3 RESULTS

**FIGURE 4:**
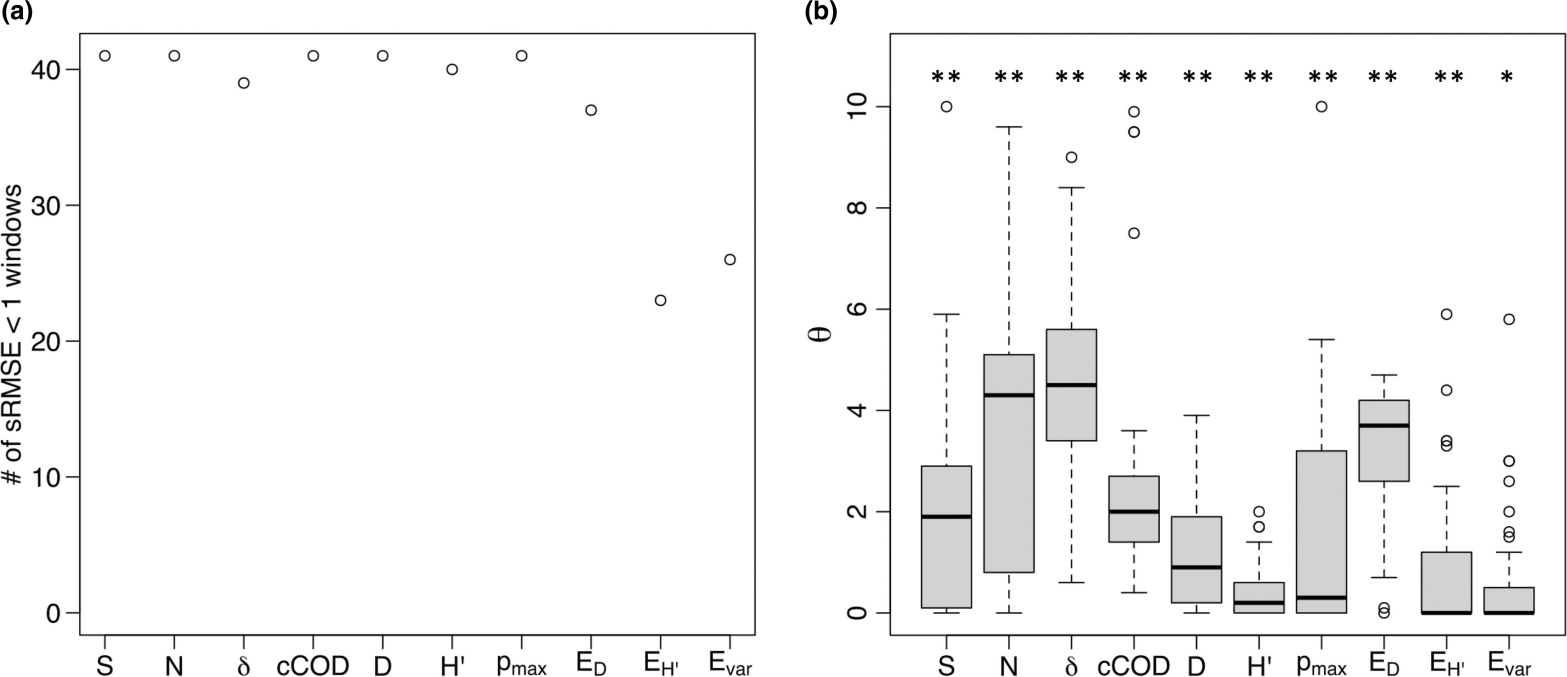
(a) Self‐prediction skills for the 10 biodiversity indicators. The vertical axis shows the windows with sRMSE of self‐prediction <1.0. (b) Boxplot showing the nonlinearity of each biodiversity indicator. The vertical axis shows the strength of nonlinearity (the parameter *θ* in the regularized S‐map). Asterisks represent the significance under the null hypothesis that “*θ* = 0 for all windows” (**p < .01 and *p < .05). Ten biodiversity indicators are species richness (S), total abundance (N), taxonomic diversity (δ), community mean of the latitudinal center of distribution (cCOD), Simpson species diversity (d), Shannon species diversity (*H*′), species dominance (*p*_max_), Simpson species evenness (*E*_D_), Shannon species evenness (*E*_H_), and Smith–Wilson species evenness (*E*_var_).

A self‐prediction analysis using simplex projection revealed that biodiversity indicators follow deterministic and nonlinear dynamics. The simplex projection successfully predicted the dynamics (i.e., sRMSE < 1) in over 90% of the windows for eight biodiversity indicators (species richness *S*, total abundance *N*, taxonomic diversity *δ*, cCOD, Simpson's diversity *D*, Shannon's diversity *H*′, species dominance *p*_max_, and Simpson's evenness *E*_D_, Figure 4a). In contrast, successful self‐prediction by simplex projection was observed in relatively low proportions of windows for Shannon's evenness *E*_H_ and Smith–Wilson evenness *E*_var_. Furthermore, a self‐prediction analysis with regularized S‐maps revealed significant nonlinearity in all biodiversity indicators (Figure 4b).

**FIGURE 5:**
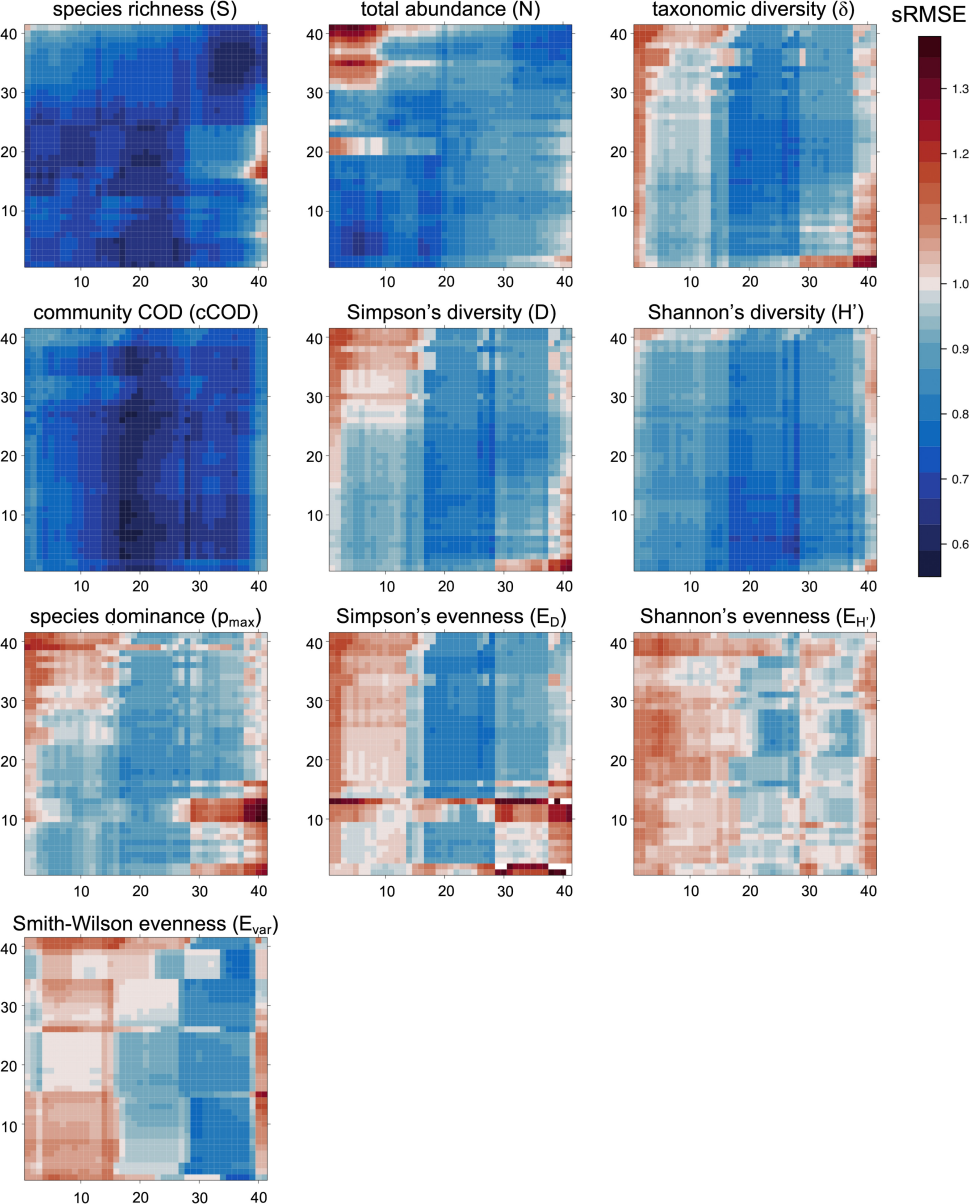
Mutual prediction matrices for all window combinations. The windows used as training data and test data for the model are displayed in each row and column, respectively.

Mutual prediction matrices suggested that the 10 indicators could be classified into three groups (Groups I–III) with similar patterns of mutual prediction (Figure 5). For Group I, mutual prediction was successful for almost all pairs (e.g., species richness *S* and cCOD). For Group II, simplex projection had good predictive performance for temporally close windows but not for temporally distant windows (e.g., Simpson's diversity *D*, taxonomic diversity (δ), and species dominance *p*_max_). For Group III, simplex projection often failed in mutual predictions, even for windows that were temporally close (e.g., Shannon's evenness $E_{H^{\prime }}$ and Smith–Wilson evenness *E*_var_).

**FIGURE 6:**
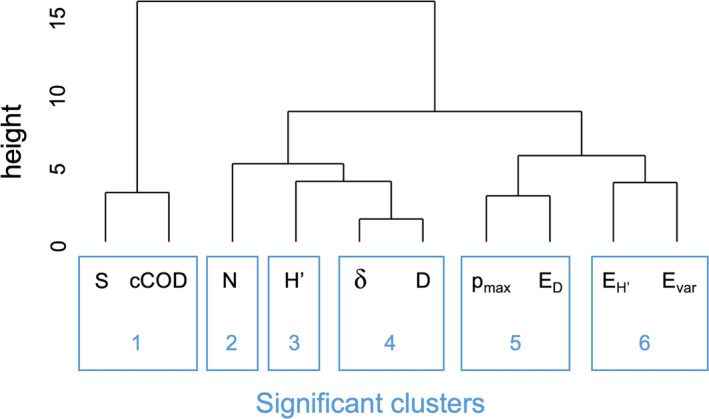
Clustering analysis based on the Euclidean distances between the prediction skill matrices using simplex projection. Ten biodiversity indicators are the same as those in Figure 4.

A hierarchical clustering analysis identified six significant clusters based on similarities in the mutual prediction matrices (Figure 6). Cluster 1 consisted of species richness *S* and cCOD; cluster 2, total abundance *N* only; cluster 3, Shannon's diversity *H′*; cluster 4, taxonomic diversity *δ* and Simpson's diversity *D*; cluster 5, species dominance *p*_max_ and Simpson's evenness *E*_D_; and cluster 6, Shannon's evenness $E_{H^{\prime }}$ and Smith–Wilson's evenness *E*_var_.

## 4 DISCUSSION

The study results showed that most biodiversity indicators had deterministic dynamics (Figure 4) and we identified three distinct groups (Groups I–III) of biodiversity indicators reflecting different dimensions of biodiversity in the fish community (Figures 5 and 6) based on similarity in environmental responsiveness. Group I consisted of species richness and community COD. This group was characterized by good mutual predictions for almost all pairs of windows (Figure 5), indicating that the biodiversity dimension reflected by these indicators is relatively insensitive to changes in temperature and other parameters during the survey period. Group II consisted mainly of species diversity indices (clusters 2–4 for total abundance, Shannon's diversity, taxonomic diversity, and Simpson's diversity). In this group, mutual prediction ability decreased for temporally distant window pairs (Figure 5), indicating that the dynamics of the biodiversity dimension reflected by these indicators changed between the first and second halves of the survey period. Group III consisted mainly of species evenness indices (clusters 5–6 for species dominance, Simpson's evenness, Shannon's evenness, and Smith–Wilson's evenness). This group had complex mutual prediction patterns (Figure 5), suggesting that the biodiversity dimensions reflected by these indicators were highly sensitive to temperature or other environmental parameters.

The environmental responsiveness of indicators in Group II indicated that the temporal fluctuations in species diversity indicators cannot be understood by a simple combination of patterns of species richness and evenness but rather reflect different dimensions of biodiversity. While species diversity dynamics showed a clear response to rapid temperature changes, species richness remained unchanged and species evenness showed complex responses. These findings are in contrast to earlier correlation‐based studies showing that species richness or species evenness share the same dimension of biodiversity with species diversity (Heino et al., 2008; Lyashevska & Farnsworth, 2012).

Concerning the pattern observed in Group III, Morris et al. (2014) found that species evenness is not consistently correlated with other biodiversity indicators across sites, despite a consistent correlation between species richness and species diversity. The authors hypothesized that the condition dependence of species evenness suggests that it represents a distinct dimension of biodiversity. The high sensitivity of species evenness identified in the present study reveals that it is highly condition dependent, supporting this hypothesis.

The dynamics of biodiversity indicators that include components of relative species richness (e.g., species diversity and evenness) were altered during the warming period, suggesting that warming alters the dynamics of relative species abundance. The mutual prediction skills of total abundance, species diversity, and taxonomic diversity decreased for window pairs spanning the first and second halves of the study period (Figure 5), suggesting that the restart of the Takahama NPP altered the dynamics of these indicators (see Appendix S2 for a more detailed analysis of the relationship between the timing of the restart of the NPP and the dynamics of biodiversity indicators). NPP‐induced warming can also be detected by the environmental responsiveness of Group III indicators. For Group III, the mutual prediction skill tended to be poor when the training data included the earliest survey period (Figure 5). Given that the Takahama NPP, previously in continuous operation, was shut down immediately before the survey period, the poor prediction skill suggests that the dynamics of Group III indicators are still in a transient state. At the survey site, the species abundance distribution also changed after warming; rare species were dominant before warming, whereas species of intermediate abundance were dominant during warming (Figure S2 in Appendix S3). This change may be related to the decrease in seasonally migrating species and increase in resident species (observed all year) after warming (Table S2 in Appendix S4). It is an intriguing question whether changes in species behavior, such as migratory/resident status, are related to changes in dynamics of biodiversity via the change in the species abundance distribution.

The classification of species richness and cCOD in the same group (Group I) may be explained by seasonal immigration and emigration/local extinction of fish species with lower cCOD. Seasonal immigration and the local extinction of tropical fish occur every year along the Sea of Japan (Nakazono, 2002) and contribute to the seasonal change in community composition in this region. Indeed, an additional analysis showed that the appearance/disappearance of most species was seasonal and that more than half of the species present in the summer were not observed in the following winter (Table S2 in Appendix S4). Furthermore, fluctuations in cCOD are due to the immigration and emigration/local extinction of fishes originating from areas at lower latitudes (Figure S3 in Appendix S4). Therefore, the assignment of species richness and cCOD to the same group at the survey site implies that these indicators reflect the same dimension of biodiversity affected by tropical fish migration.

Our results showed that biodiversity indicators can be classified into three groups that represent different biodiversity dimensions, as indicated by their environmental responsiveness. This classification can provide a basis for biodiversity conservation or management. First, the classification method can be useful when it is necessary to replace a biodiversity indicator of interest with another easily observable indicator that reflects the same dimension of biodiversity (Duelli & Obrist, 2003; Heink & Kowarik, 2010). For example, total abundance and species diversity indices (Group II) have the same environmental responsiveness in the studied community. Therefore, total abundance (or species diversity) can be a useful alternative indicator of species diversity (total abundance) for detecting community dynamics. Second, to capture more dimensions of biodiversity with as few indicators as possible (e.g., Smyth & James, 2004) in biodiversity monitoring, one should select indicators that cover the three super‐groups. Although the present grouping may be system‐specific and further studies are needed to determine generalizability of the results, the method used in this study can be applied to various communities.

## AUTHOR CONTRIBUTIONS

**Yuri Otomo:** Conceptualization (lead); data curation (lead); formal analysis (lead); investigation (lead); methodology (lead); visualization (lead); writing – original draft (lead); writing – review and editing (equal). **Reiji Masuda:** Data curation (equal); resources (lead); writing – review and editing (equal). **Yutaka Osada:** Formal analysis (supporting); investigation (supporting); methodology (supporting); writing – review and editing (equal). **Kazutaka Kawatsu:** Formal analysis (supporting); investigation (supporting); methodology (supporting); software (lead); writing – review and editing (equal). **Michio Kondoh:** Conceptualization (supporting); investigation (equal); methodology (equal); supervision (lead); writing – original draft (supporting); writing – review and editing (equal).

## FUNDING INFORMATION

YOt was supported by JST SPRING (grant number JPMJSP2114). MK and RM were supported by a JSPS KAKENHI Grant (grant number 19H05641), and MK was supported by a JSPS KAKENHI Grant (grant number 21H05315) and the Environment Research and Technology Development Fund (grant number JPMEERF20214103) of the Environmental Restoration and Conservation Agency of Japan.

## CONFLICT OF INTEREST STATEMENT

We have no conflicts of interest to declare.

## Supporting information


Appendix S1–S4

## Data Availability

All data are available at https://doi.org/10.5061/dryad.37pvmcvr1.
