# Supplementary material for: Dynamics‐based characterization and classification of biodiversity indicators
Source: Ecol Evol. 2023 Jul 6;13(7):e10271. doi: 10.1002/ece3.10271 (PMC10325886; doi:10.1002/ece3.10271)
Supplement: Supplementary file 1 — Appendix S1–S4 [file ECE3-13-e10271-s001.docx]

Appendix S1

***Consideration of the sliding window size***

To detect changes in the dynamics of biodiversity indicators, we employed the sliding window approach, in which one segment of the time series (referred to as a window) was used to predict dynamics in other segments. The window size plays a crucial role in balancing the prediction skill and sensitivity to changes in dynamics. Specifically, the self-prediction ability of each window and the sensitivity to detect the time points when mutual prediction becomes impossible are important factors to consider when selecting an appropriate window size (see Figure 3 in main text). We tested window sizes of 12, 24, 36, 48, and 60, which are multiples of 12 time points (equivalent to one year, based on monthly data collection.), to control for the effect of seasonality in the self-prediction (mutual prediction) of each biodiversity indicator.

We assessed the self-prediction skill based on the lowest standardised root mean squared error (sRMSE) (refer to Materials and Methods in the main text) when the embedding dimension E ranged from 1 to 12. The sRMSE is the root mean squared error standardised by the standard deviation of the test data. Assuming no deterministic rules, the model with simplex projection is inferior to that with no training data if sRMSE ≥ 1, as the standard deviation of the test data corresponds to the expected RMSE of a null model. We used R version 4.0.2 for analyses and the R package rEDM (version 0.7.5) for the simplex projection analysis.

The number of biodiversity indicators for which 90% of the windows had high self-prediction skills were 2, 4, 6, 8, and 7 for window sizes of 12, 24, 36, 48, and 60, respectively (Figure S1). Regardless of window size, Shannon’s evenness (*E*_D_) and Smith-Wilson evenness (*E*_var_) exhibited low proportions of windows with high self-prediction skill.


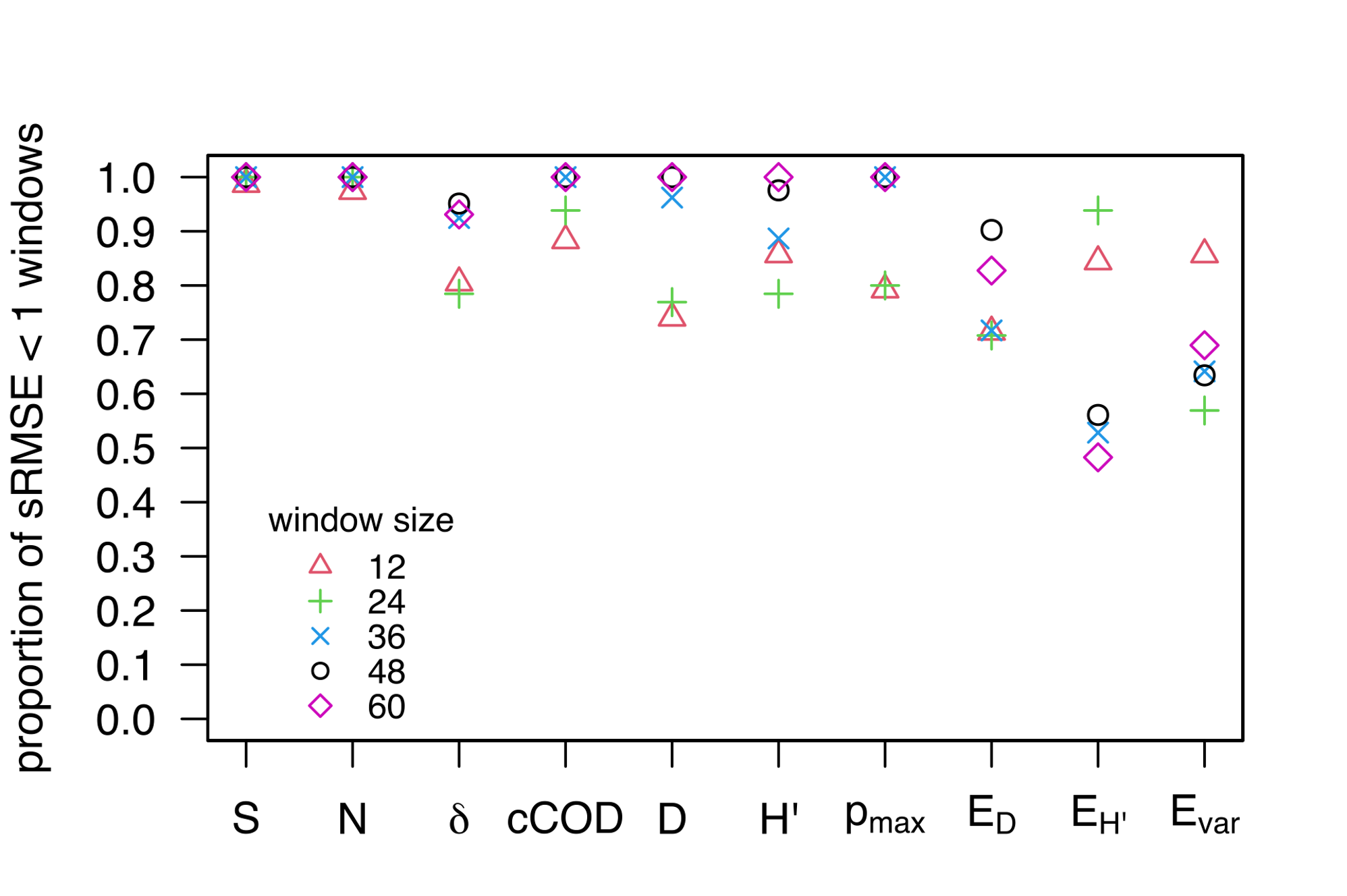


**Figure S1** Self-prediction skills for various window sizes. The vertical axis shows the proportion of windows with sRMSE of self-prediction less than 1 (i.e., self-prediction skill is better than prediction skill using null model. *S*: species richness, *N*: total abundance, δ: taxonomic diversity, cCOD: community mean of the latitudinal centre of distribution, *D*: Simpson species diversity, *H*’: Shannon species diversity, *p*_max_: species dominance, *E*_D_: Simpson species evenness, *E*_H_: Shannon species evenness, *E*_var_: Smith–Wilson species evenness.

For a window size of 48, seven biodiversity indicators showed good self-prediction skill, which is higher than those for other window sizes. We concluded that the prediction skill of each window and the sensitivity for detecting changes in dynamics would be best balanced when the window size was 48.

Appendix S2

***Relationship between changes in dynamics and the active period of the NPP***

In this study, the survey site was located 2 km from the discharge outlet of the Takahama Nuclear Power Plant (NPP). The fish community evaluate in this study was potentially impacted by warming caused by thermal discharge from the NPP. During the active periods of the NPP in the survey period (from January 2016 to March 2016 and from May 2017 to April 2019), the water temperature increased by approximately 2 °C in the study plot.

If NPP activation caused change in the dynamics of biodiversity indicators, the mutual prediction skill between two windows including the same length of NPP activity would be better. To evaluate this hypothesis, we calculated Pearson's correlation coefficients between the mutual prediction skill and the difference in active months of the NPP between the predictor and predicted window. Here, $l_{active}$ represents the number of active months in each window, and $\Delta l_{active}$ represents the difference in the number of active months between the predictor and predicted window. We assessed the significance of the correlation by comparing it with the null distribution created using surrogate data.

The null distribution was created as follows. We generated 999 discrete-time Markov chains of length 88, the length of the actual survey data used in this study. These chains had two transitional states, *S*_1_ (NPP inactive) and *S*_2_ (NPP active), which tended to have the same state continuously. A discrete-time Markov chain with an initial state was randomly determined from either *S*_1_ or *S*_2_ with a probability of 0.5. The number of times *S*_2_ occurred was set to 26 ± 5 times (26 is the number of active months of the NPP over the whole survey). The transition matrix $P=\left[ p_{ij} \right]_{(1\leq i,j\leq2)}$, where *p*_ij_ represents the transition probability of *S*_i_ to *S*_j_, was calculated from the actual survey data. The transition probabilities *p*_ij_ were calculated as follows:

|  | $p_{11}= \frac{N_{11}}{N_{11}+N_{12}}=\frac{60}{60+2}=\frac{30}{31}$ |  |
| --- | --- | --- |
|  | $p_{12}= \frac{N_{12}}{N_{11}+N_{12}}=\frac{2}{60+2}=\frac{1}{31}$ |  |
|  | $p_{21}= \frac{N_{21}}{N_{21}+N_{22}}=\frac{2}{2+23}=\frac{2}{25}$ |  |
|  | $p_{22}= \frac{N_{22}}{N_{21}+N_{22}}=\frac{23}{2+23}=\frac{23}{25}$ |  |

where *N*_ij_ is the number of transitions from *S*_i_ to *S*_j_ in the actual monitoring data. Analyses were performed in R version 4.0.2.

For each of these surrogate data, we created 41 sliding windows with a size of 48, with a step size of one time point, and calculated length of S_2_ in each window as surrogates of $l_{active}$. Thereafter, we calculated the correlation coefficients between the mutual prediction skill of actual monitoring data and $\Delta l_{active}$of each surrogate data. If the correlation coefficient between mutual prediction skill and actual $\Delta l_{active}$ was greater than 95% of the correlation coefficients using surrogate $\Delta l_{active}$, it was considered significant.

Most indicators, such as species richness, total abundance, taxonomic diversity, community mean of COD, Simpson’s species diversity, Shannon’s species diversity, species dominance, and Simpson’s species evenness, were correlated (correlation coefficient > 0.2) between the mutual prediction skill and actual $\Delta l_{active}$. For species richness, there was a significant correlation between the mutual prediction skill and actual $\Delta l_{active}$, whereas no significant correlations were found for the other indicators (Table S1).

**Table S1** Correlation coefficients of the mutual prediction skill and the actual $\Delta l_{active}$. Significance was evaluated as the percentage of larger surrogate correlations (i.e., correlations between mutual prediction skill and surrogate $\Delta l_{active}$) than the correlation coefficients of the mutual prediction skill and the actual $\Delta l_{active}$.

| Indicator | Coefficient | Significance |
| --- | --- | --- |
| species richness (*S*) | 0.775 | 0.036 |
| total abundance (*N*) | 0.698 | 0.084 |
| taxonomic diversity (δ) | 0.378 | 0.591 |
| community mean of COD (cCOD) | 0.348 | 0.215 |
| Simpson's species diversity (*D*) | 0.444 | 0.372 |
| Shannon's species diversity (*H*’) | 0.390 | 0.127 |
| species dominance (*p*_max_) | 0.583 | 0.171 |
| Simpson's species evenness (*E*_D_) | 0.276 | 0.703 |
| Shannon's species evenness (*E*_H_) | 0.061 | 0.802 |
| Smith–Wilson's species evenness (*E*_var_) | -0.023 | 0.825 |

Appendix S3

**Figure S2** Species log abundance distribution in each year. Horizontal axis shows the log abundance and vertical axis shows the number of species.

Appendix S4

***Evaluation of the hypothesis that fish migration is the major driver of species richness and community COD dynamics***

We utilised empirical fish community data to examine the similarity of environmental responsiveness among biodiversity indicators. Our analysis revealed that species richness and cCOD could be classified in the same group (Group I, see Figure 6 in main text). This classification might be explained by seasonal migration and the emigration/extinction of fish species with lower cCOD. Indeed, a previous study has reported that tropical fish (i.e. fish with lower cCOD) migrate on the Tsushima current and die out in the winter due to low water temperatures in the coastal area of the Sea of Japan (Nakazono 2002).

To investigate the impact of tropical fish migration on the dynamics of species richness and cCOD at the survey site, we conducted two analyses. First, we examined proportion of fish species observed in the summer (April–December) but not observed in the following winter (January–March) from 2012 to 2019. A consistently high proportion of fish species observed in the summer but not observed in the following winter (i.e. proportion of winter absence) every year would suggest that numerous species migrated during the summer and emigrated/died during the winter, providing indirect evidence of the impact of temporal fish immigration on species richness and cCOD dynamics. Second, we investigated whether tropical fish are more likely to immigrate and emigrate/become extinct seasonally than are fish from other regions. First, we divided the 95 observed species during the monitoring period into three groups in decreasing order of COD: Low COD (32 species, a group of species with COD below 29.5°N), Mid COD (33 species, a group of species with COD between 29.5°N and 37.5°N), and High COD (30 species, a group of species with COD above 37.5°N). We assumed that fish species in the Low COD group consisted of tropical fish. We then calculated the temporal coefficients of variation (CV) in the number of species in each COD group. Because increasing seasonal immigration would increase the CV of the number of species, we use the CV as an indicator of the number of species that immigrated seasonally and emigrated/became extinct in each COD group. If the CV in the number of species in the Low COD group is higher than those in the Mid or High COD group, it implies that tropical fish tend to immigrate more than resident fish near the survey site or from northern areas. The analyses were performed in R version 4.0.2 (R Core Team 2020).

On average, 73% of fish species were observed in the summer but not observed in the following winter during the survey period (Table S2). This result implies that most of the species observed in summer emigrate or are lost at the survey site. After restarting Takahama NPP (after the summer of 2017), the proportion of winter absence became lower than that before the NPP restarted.

|  | *S_summer_* | *S_winter absence_* | proportion of winter absence |
| --- | --- | --- | --- |
| 2012 summer | 48 | 34 | 0.71 |
| 2013 summer | 55 | 45 | 0.82 |
| 2014 summer | 49 | 41 | 0.84 |
| 2015 summer | 50 | 36 | 0.72 |
| 2016 summer | 54 | 45 | 0.83 |
| 2017 summer | 56 | 33 | 0.59 |
| 2018 summer | 56 | 33 | 0.59 |
| mean |  |  | 0.73 |

**Table S2** Proportion of fish species observed in the summer but not observed in the following winter during the monitoring period**.** *S_summer_* is the number of species observed in the summer, and *S_winter absence_* is the number of species observed in the summer but not in the following winter. The proportion of winter absence is calculated as $\frac{S_{winter absence}}{S_{summer}}$

The CV of species richness dynamics of Low COD species was 0.64, which was greater than those of Mid COD species (0.48) or High COD species (0.47). Species richness of Low COD species increased in the summer and decreased in the winter (Figure S3).


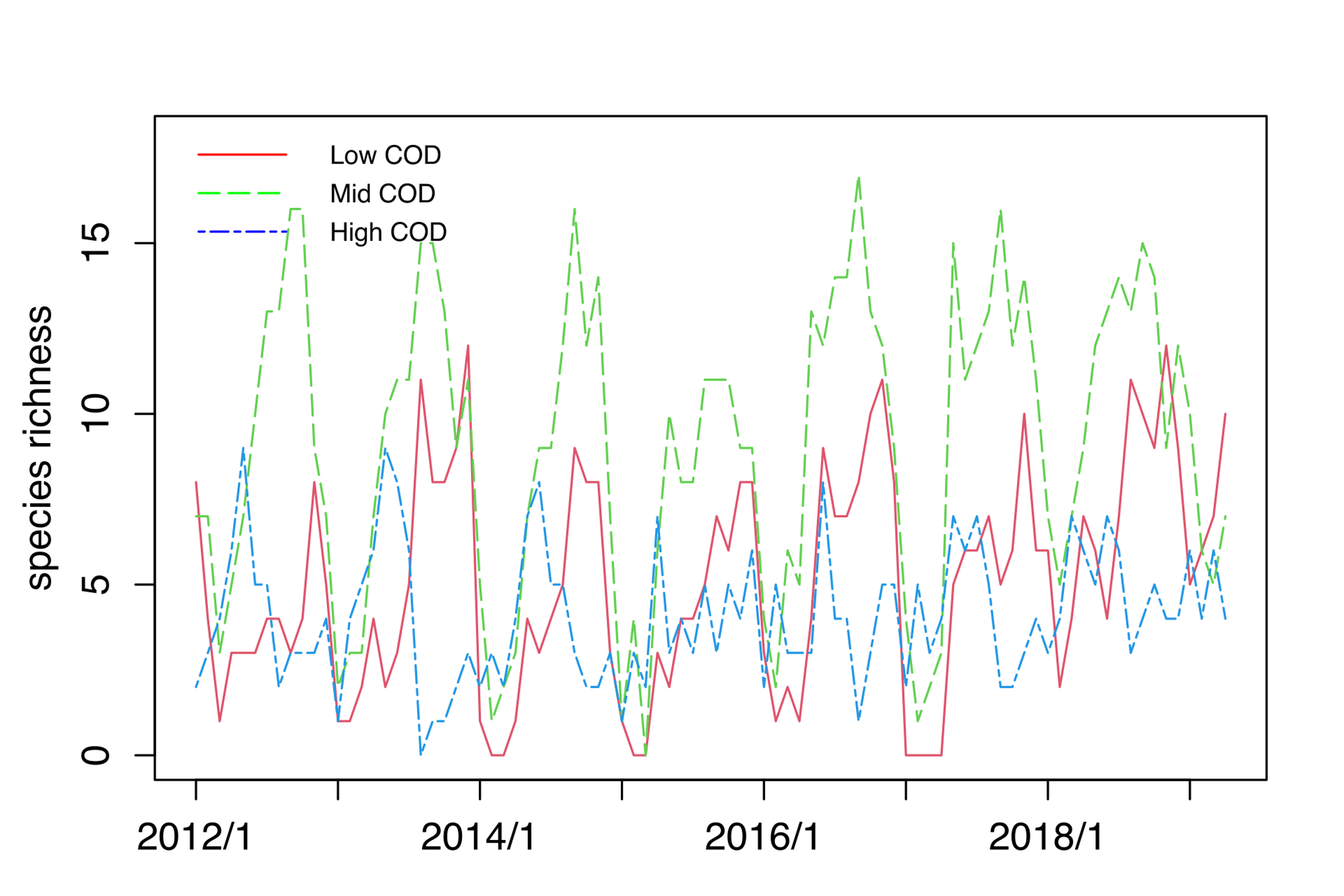


**Figure S3** Time series of species richness in the Low COD, Mid COD, and High COD groups. The horizontal axis shows the time point of each survey and the vertical axis shows species richness. The red line shows the Low COD group; green line, the Mid COD group; and blue line, the High COD group.

We found two pieces of evidence that indirectly support our hypothesis that seasonal immigration and emigration/extinctions of tropical fish are the primary cause of temporal variation in species richness and cCOD. First, a high proportion of species observed in the summer were not observed in the following winter (Table S2), suggesting that many species immigrated in the summer and emigrated/became extinct in the winter, thereby having a significant effect on species richness dynamics. Second, the species richness dynamics of tropical fish had a higher coefficient of variation (CV) than those of fish from other regions (Figure S3), implying that the proportion of species that immigrate and emigrate/become extinct seasonally was higher for tropical fish than for fish from other regions.
